# Role of substance use status in determining how caregivers of patients with opioid use disorders perceive positive aspects of caregiving and burden

**DOI:** 10.1192/j.eurpsy.2023.1193

**Published:** 2023-07-19

**Authors:** P. Saha, S. Saha, A. Singh, S. Sarkar, G. S. Kaloiya

**Affiliations:** Department of Psychiatry and National Drug Dependence Treatment Centre, All India Institute of Medical Sciences, New Delhi, India

## Abstract

**Introduction:**

Substance dependence affects an individual as well as the family and is considered as a complex biopsychosocial phenomenon. Family members can act as a social and emotional support in the treatment engagement and recovery of the patient with substance use disorder. Caregiving is a multidimensional construct. Caregiving process to an individual with substance use disorder can help in either positive or negative outcome and is often challenging. Positive aspects of caregiving has gathered some attention in mental health literature in recent past, data for the same is limited across substance use disorder.

**Objectives:**

To determine whether substance use status is associated with differences in positive aspects of caregiving and burden among the caregivers of patients with opioid use disorders.

**Methods:**

A cross-sectional observational study with purposive sampling was used to recruit 199 caregivers of patients with opioid use disorders. The sample was divided based upon the current substance use status of the patients. Scale for Positive Aspects of Caregiving Experience (SPACE) and Family Burden Interview Schedule (FBIS) were used to assess positive aspects of caregiving and family burden respectively

**Results:**

The study included 199 caregivers of patients with opioid use disorder. Table 1 describes the socio-demographic profile of the patients and caregivers. Of 199 caregivers recruited, 135 (67.8%) reported that the patient was using opioids, while 64 (32.2%) reported that the patient was abstinent on treatment. The mean SPACE domain score of caregivers abstinent on treatment was highest for motivation for caregiving role (2.73 versus 1.76) followed by self-esteem and social aspect of caring (2.42 versus 1.87), caregiver satisfaction (2.41 versus 1.29) and caregiving personal gains (2.40 versus 1.45). Details of SPACE domain score and FBIS are depicted in table 2. It was seen that caregivers of patients currently abstinent on treatment experienced greater positive aspects of caregiving (SPACE mean score 128.3 versus 80.1, t = 9.383, p <0.001), and lesser burden (FBIS mean score 13.4 versus 29.3, t = 10.419, p <0.001). Overall the mean SPACE domain score had a negative correlation with FBIS (r = -0.57, p<0.001).

**Image:**

**Figure:**
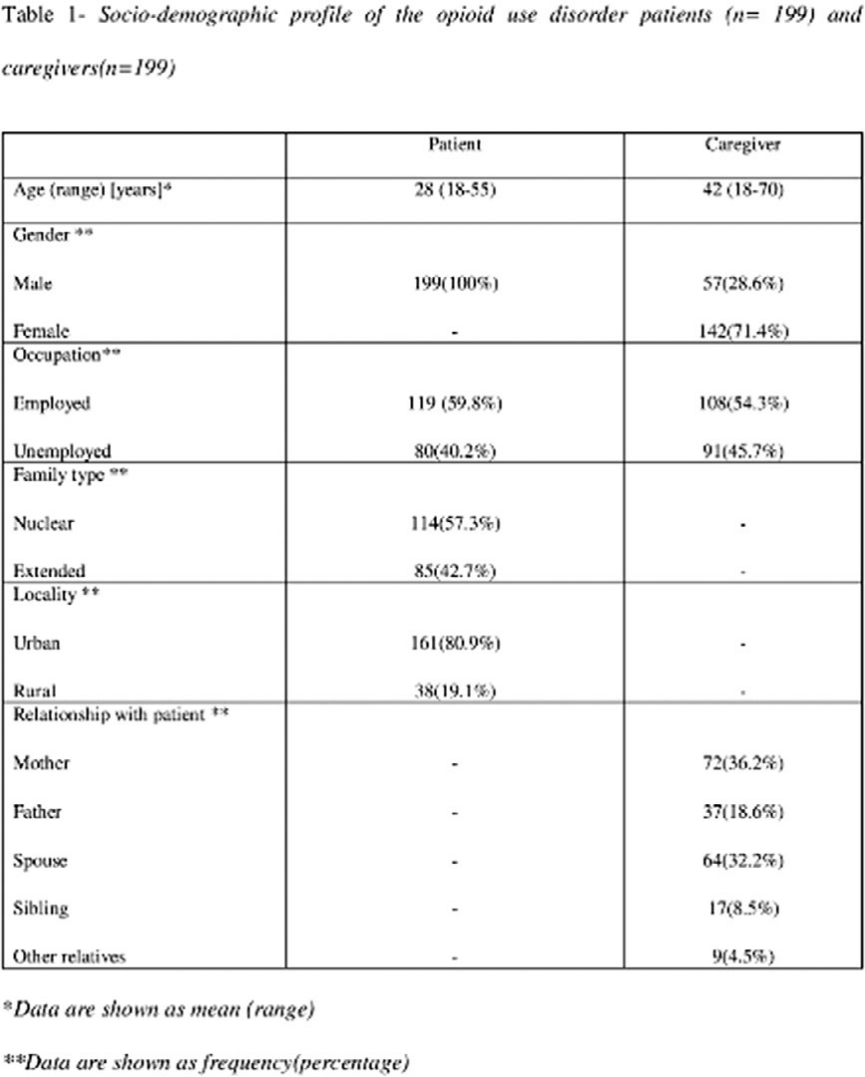

**Image 2:**

**Figure:**
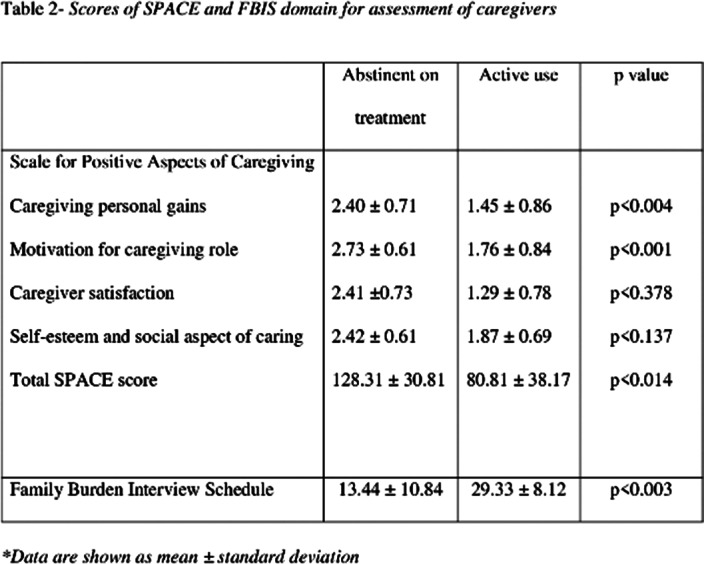

**Conclusions:**

In our study it was found that caregivers of patients who are currently abstinent on treatment experience lower burden of care, and also experience greater positive aspects of caregiving. Clinicians should be aware of the caregiver experiences as well as they engage both patients and caregivers in the treatment process.

Keywords: Positive Aspects of Caregiving Experience; Family Burden; Caregivers; Opioid; Substance.

**Disclosure of Interest:**

None Declared

